# Proposed Update to the Taxonomy of Pestiviruses: Eight Additional Species within the Genus *Pestivirus*, Family *Flaviviridae*

**DOI:** 10.3390/v13081542

**Published:** 2021-08-04

**Authors:** Alexander Postel, Donald B. Smith, Paul Becher

**Affiliations:** 1 Institute of Virology, University of Veterinary Medicine, 30559 Hannover, Germany; alexander.postel@tiho-hannover.de; 2 Nuffield Department of Experimental Medicine, University of Oxford, Peter Medawar Building, South Parks Road, Oxford OX1 3SY, UK; donald.smith@ndm.ox.ac.uk

**Keywords:** *Flaviviridae*, pestivirus, taxonomy, species, genetic distances, phylogenetic analysis

## Abstract

Pestiviruses are plus-stranded RNA viruses belonging to the family *Flaviviridae.* They comprise several important pathogens like classical swine fever virus and bovine viral diarrhea virus that induce economically important animal diseases. In 2017, the last update of pestivirus taxonomy resulted in demarcation of 11 species designated *Pestivirus* *A* through *Pestivirus K*. Since then, multiple new pestiviruses have been reported including pathogens associated with disease in pigs or small ruminants. In addition, pestivirus sequences have been found during metagenomics analysis of different non-ungulate hosts (bats, rodents, whale, and pangolin), but the consequences of this pestivirus diversity for animal health still need to be established. To provide a systematic classification of the newly discovered viruses, we analyzed the genetic relationship based on complete coding sequences (cds) and deduced polyprotein sequences and calculated pairwise distances that allow species demarcation. In addition, phylogenetic analysis was performed based on a highly conserved region within the non-structural protein NS5B. Taking into account the genetic relationships observed together with available information about antigenic properties, host origin, and characteristics of disease, we propose to expand the number of pestivirus species to 19 by adding eight additional species designated *Pestivirus* *L* through *Pestivirus* *S*.

## 1. Introduction

Pestiviruses are RNA viruses belonging to the family *Flaviviridae* and comprise pathogens of livestock like classical swine fever virus (CSFV) and bovine viral diarrhea virus (BVDV) that cause diseases of outstanding economic relevance notifiable to the World Organization for Animal Health (OIE) [1,2,3]. In early 2000, the genus *Pestivirus* included the four species *Border disease virus*, *Bovine viral diarrhea virus 1*, *Bovine viral diarrhea virus 2*, and *Classical swine fever virus*; the ‘giraffe’ pestivirus was then unclassified [4,5]. Subsequently, additional pestiviruses were discovered in domestic and wild animals, including HoBi-like pestiviruses from cattle and buffalo [6,7]; pronghorn antelope virus from a diseased antelope in the USA [8,9]; the unique Bungowannah virus isolated from a pig farm in Australia [10]; Aydin-like pestiviruses from sheep and goats in Turkey [11,12], recently also identified in cattle [13]; and atypical porcine pestivirus that is today well-known as a major cause of congenital tremor in newborn piglets worldwide [14,15,16,17,18]. In many cases, these new pestiviruses are associated with abortion, still birth, or health problems of offspring. Due to the ability of pestiviruses to establish persistent infections after intrauterine infection, persistently infected hosts (including wild animals) can play an important role as virus reservoirs [19]. For many decades, it was believed that pestiviruses only infect cloven-hoofed animals. However, in the past decade, several metagenomics studies have discovered pestivirus genomes in non-ungulate host species; these sequences are rarely associated with virus isolation, and associations with disease are unknown [20,21,22,23,24,25]. It will be of great interest to study the host range, prevalence, geographic distributions, and other aspects of the biology of these novel viruses and to elucidate their potential as newly emerging pathogens in farm and wild animals. Due to the increasing number of diverse pestiviruses, the taxonomy of the genus *Pestivirus* was revised in 2017 [26] based on nucleotide or amino acid sequence distances of complete coding sequences (cds), in combination with antigenic differences, natural host range, and pathology [27]. In consequence, pestiviruses are currently classified into 11 species designated *Pestivirus A* through *Pestivirus K* (Table 1).

In the present study, the same criteria for species demarcation [26] were applied to classify recently discovered pestiviruses resulting in a proposed update of the taxonomy of the genus *Pestivirus*. Based on a systematic analysis of pairwise genetic distances among the complete coding sequences of representatives of the 11 established species and recently reported pestiviruses, together with the available data characterizing these viruses, we propose that eight additional species (*Pestivirus L* through *Pestivirus S*) should be added to the genus *Pestivirus*.

## 2. Materials and Methods

Analysis of the genetic relationship between different pestiviruses was performed as previously proposed by the *Flaviviridae* Study Group of the ICTV [26]. Briefly, cds and deduced polyprotein sequences were aligned with MUSCLE (Neighbour joining algorithm), and pairwise distances (p-distances) were calculated with the tools implemented in MEGA X (Version 10.0.5) [36]. The previously established set of 67 representative sequences of the approved species *Pestivirus A* through *Pestivirus K* was used [26]. For atypical porcine pestivirus (APPV, *Pestivirus K*), the number of available complete coding sequences has increased from six to 69, and in order to reflect this increased diversity, eight genetically distinct sequences were added to the previously used sequence data set (Table 2).

To identify novel pestivirus sequences, a GenBank search was performed using the search term ‘Pestivirus 5000[SLEN]:20000[SLEN]’ on 8 January 2021 as described earlier [26]. The search resulted in 785 sequences entries. The dataset was reduced by discarding entries containing names of established species and artificial sequences e.g., related to patents. One sequence was excluded that was identified in a metagenomics approach investigating viromes of ticks. Although this virus is most closely related to pestiviruses, it shares less than 30% identity to the highly conserved pestiviral non-structural proteins NS3 and NS5 and thus likely represents a new genus within the family *Flaviviridae* [37]. In total, 17 novel pestivirus sequences were identified with a length of more than 5000 nucleotides (Table 3). In addition, we added the complete genome sequences of the pestivirus isolate 92019/2007/AG (GenBank MZ664274) and the closely related cds of 70282/2007/EN (GenBank MZ664273) obtained from sheep and goat in Sicily, Italy (Table 3). Previous analysis of partial 5′ NTR and complete N^pro^ coding sequences showed that both of these virus isolates belong to a group of Tunisian sheep pestivirus-like viruses (TSV) [38]. Establishment of the complete genomic sequences of the two TSV isolates is described elsewhere [39].

**Figure 1:**
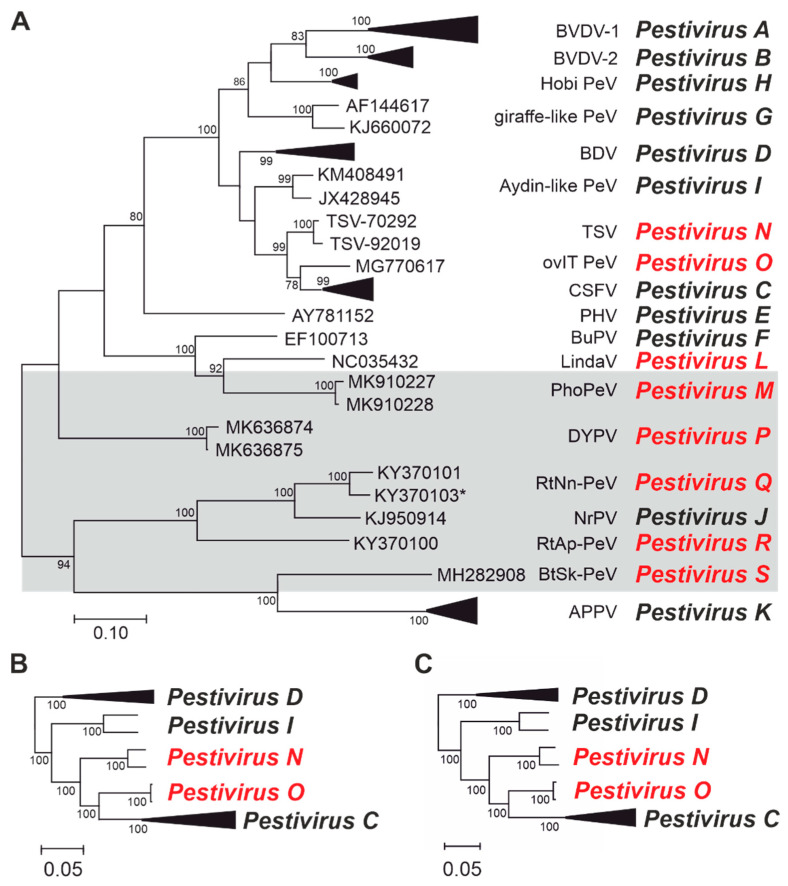
Phylogenetic characterization of known pestiviruses. (**A**) Construction of the Maximum likelihood tree was based on the conserved partial NS5B peptide sequences corresponding to positions 3312–3837 in the polyprotein of BVDV-1 (*Pestivirus A)* reference strain SD-1. With one exception (KY370103, indicated by asterisk), the sequences were derived from complete coding sequences. Core data set was used as established earlier and modified as described in Materials and methods [26]. Given are GenBank accession numbers and abbreviations of virus names as listed in Table 1 and Table 3. The established (*Pestivirus A* through *Pestivirus K*; black) and newly proposed (*Pestivirus L* through *Pestivirus S*; red) pestivirus species are indicated next to the tree. Pestivirus sequences identified in non-ungulate hosts are highlighted (grey shaded box). For clarity, the clades for viruses in the species *Pestivirus A*, *Pestivirus B*, *Pestivirus C*, *Pestivirus D*, *Pestivirus H,* and *Pestivirus K* are collapsed. For statistical analysis, 1000 bootstrap repetitions were performed. Only bootstrap values >70% are shown. A subset of pestivirus species closely related to TSV (*Pestivirus N*) and ovIT PeV (*Pestivirus O*) was analyzed separately by Maximum likelihood analyses for complete coding sequences (**B**) and polyprotein sequences (**C**) to compare the topologies of the trees.

For phylogenetic analysis, amino acid sequences of a conserved region of NS5B corresponding to positions 3312–3837 in the polyprotein of BVDV-1 (*Pestivirus A*) reference strain SD-1 were compared (Figure 1A). Maximum likelihood trees were generated using the complete cds (Figure 1B) and polyprotein amino acid sequences (Figure 1C). Phylogenetic analysis of the NS5B region served as one criterion for the last update of pestivirus taxonomy, which resulted in the approval of the 11 species [26]. Multiple sequence alignment was performed using MUSCLE, and phylogenetic trees were generated by maximum likelihood applying the JJT model as implemented in MEGA X [36]. To reduce the size of the tree, two redundant sequences were discarded (GenBank MK618726, MK910229) and clades including multiple sequences of BVDV-1 (*Pestivirus A*), BVDV-2 (*Pestivirus B*)*,* CSFV (*Pestivirus C*), BDV (*Pestivirus D*)*,* HoBi-like pestiviruses *(Pestivirus H*), and APPV (*Pestivirus K*) were collapsed. Phylogenetic analyses based on the complete cds and polyprotein amino acid sequences were additionally performed with a reduced sequence set of CSFV (*Pestivirus C*), BDV (*Pestivirus D*), Aydin-like pestiviruses (*Pestivirus I*), TSV, and novel ovine pestiviruses from Italy (ovIT PeV) (Figure 1B,C).

## 3. Results and Discussion

### 3.1. Update of the Reference Data Set

The dataset previously used for pestivirus species demarcation was adjusted as the number of sequences available for APPV (*Pestivirus K*) has increased significantly, revealing greater genetic variation. Comparison of the cds of six APPV reference sequences used for the last update of pestivirus taxonomy in 2017 revealed p-distances on genomic nucleotide and amino acid level of <0.13 and <0.06, respectively [26]. Genetic diversity in the updated APPV (*Pestivirus K*) data set increased these to <0.20 and <0.10, respectively, similar to that observed for CSFV (*Pestivirus C*) (Table 4). It has been previously reported that the maximum genomic nucleotide and amino acid p-distances between members of the same species did not exceed 0.24 and 0.15, respectively, with the highest diversity observed within BVDV-1 (*Pestivirus A*) and BDV (*Pestivirus D*) [26] (Table 4). The minimum nucleotide and amino acid p-distances between members of different species are >0.28 and >0.19 (between members of *Pestivirus I* and *Pestivirus D*, Table 5). In all cases, p-distances of the conserved NS5B peptide are less than 0.13 between members of the same species and exceed 0.13 between members of different species.

### 3.2. Recently Identified Pestiviruses Represent Novel Species

In principle, species epithets for novel species will be capital letters given chronologically. As in this case where several species are being proposed at the same time, this allows some flexibility. Like in the last update of taxonomy, letters were chosen that evoke an association with the respective virus.

#### 3.2.1. Linda Virus

Shortly after the last update of pestivirus taxonomy introducing the species names *Pestivirus A* through *Pestivirus K*, a novel pestivirus was discovered in a commercial pig farm in Austria in association with congenital tremor in newborn piglets [40]. This virus was successfully isolated on established porcine cell lines and named LINDA virus (LindaV). The complete genome sequence (GenBank NC035432) reveals that LindaV is genetically most closely related to Bungowannah virus (BuPV, *Pestivirus F*; p-distance 0.35, Table 5), which was isolated from diseased pigs in a large pig holding in Australia in 2005 [10]. Phylogenetic analysis of NS5B_3312–3837_ peptides of the reference set of pestivirus sequences confirmed the genetic relationship of LindaV to BuPV (Figure 1). The observation that nucleotide and amino acid distances between LindaV and BuPV (*Pestivirus F*) are greater than those observed between members of different existing pestivirus species (Table 5) supports the proposal of Kiesler et al. [42] that LindaV is a member of a new pestivirus species. This being the first pestivirus to be described after the establishment of *Pestivirus K* and since the virus is called LINDA virus, we propose to name this species *Pestivirus L*. Consistent with the conclusion that it belongs to a novel species, LindaV can induce congenital tremor, which has not been reported in BuPV infected piglets [10,40]. Moreover, it has been reported that antisera obtained from pigs after acute infection with LindaV do not show cross-neutralization against BVDV-1 (*Pestivirus A*), BVDV-2 (*Pestivirus B*), CSFV (*Pestivirus C*), BDV (*Pestivirus D*), Giraffe-like pestivirus (*Pestivirus G*) or HoBi-like pestivirus (*Pestivirus H*) [42]. However, members of these different pestivirus species can be detected by the broadly cross-reactive E2-specific monoclonal antibody 6A5. This mab is also able to detect LindaV infections, providing evidence for a commonly shared epitope [40]. Similarly, a porcine convalescence serum that neutralized BuPV (ND_50_ 1/3200) also efficiently neutralized LindaV (ND_50_ 1/1600) in a virus neutralization test [24,42]. These cross-reactivities may cause problems in serological discrimination of pestiviruses.

#### 3.2.2. Phocoena Pestivirus

In 2019, a novel pestivirus, Phocoena pestivirus (PhoPeV), was discovered in harbor porpoises (*Phocoena phocoena*) [22], the virus replicating in bovine (MDBK) and porcine (PK-15) cells. Genome fragments of different pestiviruses present in samples from harbor porpoises collected at the North Sea coast show a diversity of less than 10% at the nucleotide level [22]. In addition, three very similar complete genome sequences have been obtained (GenBank MK910227-29), including one that harbors a 180 nucleotide insertion within the p7-NS2 coding region (GenBank MK910229). PhoPeV is most closely related to LindaV (*Pestivirus L*) and—more distantly—to BuPV (*Pestivirus F*, Figure 1) with genomic p-distances at the nucleotide and amino acid level of >0.37 and >0.36, respectively, and amino acid p-distances of >0.24 for NS5B_3312–3837_ (Table 5), consistent with PhoPeV representing an additional pestivirus species. Being the first pestivirus discovered in a marine mammal, we propose to designate this species *Pestivirus M.* Uniquely among pestiviruses, all known genome sequences of PhoPeV lack the pestivirus-specific N^pro^ encoding sequence, possibly because of differences in the innate immune system of cetaceaen host species [22]. Sequences of the E^rns^ protein, a second protein unique to pestiviruses, are present in the genomes of PhoPeV isolates. PhoPeV is apparently highly prevalent in the harbor porpoise population of the Dutch North Sea, providing evidence for long lasting or persistent infection as also known for biologically well characterized pestiviruses like BVDV-1 (*Pestivirus A*) and CSFV (*Pestivirus C*) [22]. So far, no information on antigenic relatedness between PhoPeV (*Pestivirus M*) and members of other pestivirus species has been reported.

#### 3.2.3. Pangolin Pestivirus

Pestivirus sequences have been recently discovered in fatally diseased pangolins in China and named Dongyang pangolin virus (DYPV) [21]. Two very similar complete genome sequences have been obtained, but no virus isolate is available. Comparative analysis of complete coding and polyprotein sequences revealed genomic nucleotide and amino acid p-distances between DYPV and other pestiviruses of >0.41 and >0.47, respectively (Table 5). Interestingly, nucleotide p-distances are slightly lower than amino acid p-distances. Amino acid distances for NS5B_3312–3837_ are >0.33 (Table 5), consistent with DYPV representing an additional pestivirus species. As a virus belonging to this species was first detected in samples from pangolins, we propose to name it *Pestivirus P*.

#### 3.2.4. Bat Pestiviruses

Genomic sequences of five different pestiviruses (GenBank MH282908—MH282911, JQ814854) have been detected in bats in China (23, 25), these being most closely related to APPV (*Pestivirus K*). Four sequences, including a complete cds (GenBank MH282908, BtSk-PeV-1/GX2017) obtained from a bat of the species *Scotophilus kuhlii*, are very similar to each other, while one partial sequence from a bat of the species *Rhinolophus affinis* (GenBank JQ814854) is significantly different with nucleotide p-distances of 0.34–0.36 for the 680 nucleotide region corresponding to positions 4742–5421 of GenBank MH282908, much higher than among the *Scotophilus kuhlii* virus sequences (0.01–0.05). A similar divergence was observed for the region corresponding to positions 2444–7452 of GenBank MH282908 (distances of 0.34–0.36 compared to <0.02 among three of the *Scotophilus kuhlii* virus sequences). Although these results are consistent with the bat pestivirus sequences belonging to two additional species, only one of these sequences is a complete genome sequence (MH282908, from *Scotophilus kuhlii*). Comparisons of this sequence with representatives of existing pestivirus species (Table 5) are consistent with this representing an additional species. Due to the first description of the virus from this species in samples from *Scotophilus* bats, it is proposed to use the designation *Pestivirus S* for this novel species.

#### 3.2.5. Rodent Pestiviruses

Rodent pestivirus sequences were first reported in a Norway rat (*Rattus norvegicus*) in New York City in 2014. This novel pestivirus was designated Norway rat pestivirus (NrPV) and assigned to the species *Pestivirus J* [20]. More recently, two complete genome sequences of pestiviruses were reported from the Chinese rodents *Niviventer niviventer* (GenBank KY370101) and *Apodemus peninsulae* (GenBank KY370100) [23,24] with minimum nucleotide and amino acid p-distances to the Norway rat pestivirus of 0.30 and 0.22, and even higher p-distances (0.4 and 0.42) between each other, with similar results for the NS5B_3312–3837_ region (0.16 to the rat pestivirus, 0.32 between each other). We propose that these represent two additional species. The species names *Pestivirus Q* (GenBank KY370101) and *Pestivirus R* (GenBank KY370100) are proposed with the letter “R” referring to the rodent host and “Q” the preceding letter in the alphabet. Additional partial sequences obtained from Chinese rodents (GenBank KY370099, KY370102, KY370103) are very similar and belong to the species *Pestivirus Q*. Only in one case (GenBank KY370103) does the available sequence cover also the region encoding for the conserved NSB peptide, which has an amino acid p-distance of only 0.06 to the most closely related member of *Pestivirus Q*. The viruses NrPV (*Pestivirus J*), the novel rodent pestiviruses (*Pestivirus Q*, *Pestivirus R*), the bat pestivirus (*Pestivirus S*), and APPV (*Pestivirus K*) form one monophyletic clade (Figure 1). Within this clade, antigenic relationships to members of other pestivirus species have been described only for APPV (*Pestivirus K*) [43]. The association between virus infection and disease or pathology is known for APPV but not for other members of this clade [15,17].

#### 3.2.6. Tunisian Sheep-Like Viruses

Pestiviruses were discovered 20 years ago in diseased small ruminants in Tunisia [44,45]. These viruses were designated as Tunisian sheep-like viruses (TSV) or Tunisian small ruminant viruses (TSRV) and were subsequently also found in France and Italy [38,46,47]. Comparison of partial sequences showed that TSV is most closely related to CSFV (*Pestivirus C*) and Aydin-like pestiviruses (*Pestivirus I*) [12,38] (Figure 1). Phylogenetic analyses based on partial genome sequences suggested that TSV probably represents a novel pestivirus species [6,38]. Complete coding sequences were recently established for two TSV isolates [39], and comparative analysis of these sequences revealed nucleotide and amino acid p-distances of >0.24 and >0.16 to CSFV and >0.26 and >0.18 to Aydin-like pestiviruses, respectively. These p-distances are slightly higher than the respective maximum nucleotide and amino acid p-distances of <0.24 and <0.15 observed within BDV (*Pestivirus D*, Table 4). Analysis of partial NS5B_3312–3837_ sequences revealed p-distances of >0.13 between TSV and Aydin-like pestiviruses (*Pestivirus I*) and distances of >0.09 between TSV and CSFV isolates (*Pestivirus C*), the latter being lower than observed between members of existing species (Table 5).

According to the criteria for species demarcation defined by the ICTV *Flaviviridae* study group, besides the genetic relationship of viruses, the host species and the ability to cause disease are also considered in the definition of species [27]. Although TSV is genetically closely related to CSFV (*Pestivirus C*), the natural host range of TSV is restricted to small ruminants and it has not been identified in porcine hosts. Moreover, after experimental infection of pigs, no CSF-like symptoms were observed [39]. Taking into account these biological properties and the calculated p-distances of complete coding sequences and polyprotein sequences, it is proposed that TSV represents a separate species designated as *Pestivirus N*.

#### 3.2.7. Ovine/Italy Pestiviruses

An additional group of pestiviruses capable of inducing Border disease-like symptoms has recently been discovered in small ruminants from Italy [41]. Analysis of the complete genome sequences of these viruses revealed a close relationship to a short partial 5′ NTR sequence of a pestivirus identified in 1999 in a sheep flock in Northern Spain and associated with abortion, stillbirth, and weak lambs [48]. Comparative analysis of complete coding sequences and polyprotein sequences showed that the ovine Italian pestivirus isolates (ovIT PeV, in literature also abbreviated OVPV) are closely related to CSFV (*Pestivirus C*) and TSV (*Pestivirus N*) [41]. Nucleotide and amino acid p-distances are >0.22 and >0.14 to CSFV (*Pestivirus C*) and 0.23 and >0.14 to TSV (*Pestivirus N*), respectively (Table 4), similar to the distances of <0.22 and <0.15 observed between sequences of BVDV-1 (*Pestivirus A*) or <0.24 and <0.15 between sequences of BDV (*Pestivirus D*). For the conserved NS5B_3312–3837_ peptide sequences, p-distances between ovIT isolates and CSFV (*Pestivirus C*) are higher than 0.10 and distances between ovIT isolates and TSV (*Pestivirus N*) are 0.11. Thus, nucleotide and amino p-distances of complete or partial genome sequences do not clearly support the classification of ovIT into an additional species. Similarly, phylogenetic analysis of these genome regions reveal that ovIT shares a branch with CSFV (*Pestivirus C,* Figure 1). However, on the basis that there is no evidence for ovIT natural infection of pigs, we propose to classify ovIT pestiviruses as an additional pestivirus species. As these pestiviruses were first isolated from ovine hosts and are closely related to TSV (*Pestivirus N*), we propose to assign consecutive letters and name the species *Pestivirus O*.

## 4. Conclusions

In this study, we systematically analyzed recently reported sequences of novel pestiviruses. As a result, we propose to establish eight additional species (*Pestivirus L*, *Pestivirus M*, *Pestivirus N*, *Pestivirus O*, *Pestivirus P*, *Pestivirus Q*, *Pestivirus R,* and *Pestivirus S*), expanding the number of pestivirus species to 19. Most of the new viruses are genetically very distinct from members of the previously approved species *Pestivirus A* through *Pestivirus K*. Thus, the calculation of p-distances between complete coding nucleotide sequences and between complete viral polyprotein sequences is very useful for species demarcation. When analyzing p-distances based on genome sequences, the identification of genetic insertions and other genomic rearrangements as well as manual editing can be required [26]; such genomic alterations frequently occur in the genomes of cytopathic pestiviruses but may also be present in the genomes of non-cp pestiviruses [49]. In this study, phylogenetic analysis of a highly conserved part of NS5B (NS5B_3312–3837_) was consistent with that observed for complete genome sequences, and NS5B_3312–3837_ p-distances were useful in the demarcation of pestivirus species. An advantage of using NS5B_3312–3837_ is that this region is not prone to large-scale insertion or deletion and so no additional editing of sequences is required. However, analysis of this region is less useful in demarcating CSFV (*Pestivirus C*)*,* TSV (*Pestivirus N*), and ovIT PeV (*Pestivirus O*).

The genetic and antigenic similarity of CSFV, TSV, and ovIT isolates complicates the demarcation of species in the genus *Pestivirus*. A close genetic and antigenic relationship between the ovIT isolates (*Pestivirus O*) and CSFV (*Pestivirus C*) has been recently reported and may cause problems in CSF molecular and serological diagnosis if the ovine pestiviruses are ever shown to be able to infect porcine hosts under natural conditions [50,51]. Genetic analysis based on p-distances was not suited to demarcate the species *Pestivirus N* and *Pestivirus O*, which are closely related to each other and to CSFV (*Pestivirus C*) as calculated distances lie in a range that do not show a clear affiliation. Instead, topology of phylogenetic trees, host range, and the clinical signs are considered the most important criteria. Phylogenetic trees of cds, polyprotein, and conserved NS5B sequences clearly showed that ovIT PeV (*Pestivirus O*) shares a common branch with CSFV (*Pestivirus C*) but is distinct from CSFV (Figure 1).

Consequently, the topology of phylogenetic trees provides a strong argument to recognize ovIT isolates and TSV as members of two different species being also different to the established species *Pestivirus C*. However, it can be speculated that further pestiviruses may exist that will fill up the gaps between these branches in the phylogenetic tree. Furthermore, a clear discrimination to CSFV (*Pestivirus C*) might become challenging if viruses similar to TSV (*Pestivirus N*) and ovIT PeV (*Pestivirus O*) are found to cross the species barrier and efficiently infect and replicate in porcine hosts under natural conditions. In this context, it is important to emphasize that, despite close genetic and antigenic relatedness of CSFV (*Pestivirus C*)*,* TSV (*Pestivirus N*), and ovIT PeV (*Pestivirus O*), clear differences exist in host specificity and clinical disease induced by both ruminant pestiviruses in comparison to CSFV (*Pestivirus C*) [39,51]. TSV (*Pestivirus N*) was discovered in 1995 and since that time, no natural infections have been reported in porcine hosts. Moreover, despite retrospective evidence for the presence of ovIT PeV (*Pestivirus O*) in small ruminants in the late 1990s, these viruses have also never been found in pigs. Two recent studies demonstrated that these ruminant pestiviruses are able to infect pigs under experimental conditions, but viral replication was very limited. In addition, replication of TSV (*Pestivirus N*) on porcine cells was demonstrated to be rather inefficient *in vitro*. Moreover, these ruminant pestiviruses were either not able to induce disease (in the case of TSV) or induced only mild, unspecific clinical signs and pathological alterations (in the case of ovIT isolates) that resemble the outcome of horizontal infections of pigs with other ruminant pestiviruses, e.g., BVDV-1 (*Pestivirus A*) and BDV (*Pestivirus D*) [39,51].

Taken together, the topology of phylogenetic trees, host specificity, and induced clinical signs support the proposed classification of TSV and ovIT virus isolates as two novel pestivirus species (*Pestivirus N* and *Pestivirus O*). In addition, regulatory aspects implicated in the control of CSF require a clear differentiation of CSFV from other pestiviruses and emphasize the importance of surveillance as well as molecular, serological, and biological characterization of established and novel pestiviruses circulating in domestic and wild animals.

## Figures and Tables

**Table 1:** Established pestivirus species and reference sequences.

Species	Virus Name	Abbreviation ^1^	Host Species	Reference Isolate	Reference Sequence		
					GenBank	Length [b]	Reference
*Pestivirus A*	bovine viral diarrhea virus-1	BVDV-1	*Bos* sp., *Ovis* spp., *Capra* spp., *Artiodactyla*	SD-1 NADL (cp)	M96751 M31182	12,308 12,573	[28,29]
*Pestivirus B*	bovine viral diarrhea virus-2	BVDV-2	*Bos* sp., *Ovis* spp., *Capra* spp., Artiodactyla	XJ-04 890	FJ527854 U18059	12,284 12,513	Gen-Bank, [30]
*Pestivirus C*	classical swine fever virus	CSFV	*Sus scrofa*	Alfort/187	NC038912	12,298	[31]
*Pestivirus D*	border disease virus	BDV	*Ovis* spp., *Capra* spp., *Artiodactyla*	X818	NC003679	12,333	[32]
*Pestivirus E*	pronghorn antelope pestivirus	PHV	*Antilocapra americana*		NC024018	12,273	[8]
*Pestivirus F*	porcine pestivirus	BuPV	*Sus scrofa*	Bungowannah	NC023176	12,656	[10]
*Pestivirus G*	giraffe pestivirus	-	*Bos taurus*, *Giraffa camelo-pardalis*	PG-2 H138 (cp)	KJ660072 NC003678	12,264 12,602	[33,34]
*Pestivirus H*	HoBi-like pestivirus	HoBi	*Bos* sp.	Th/04_ KhonKaen	NC012812	12,337	[35]
*Pestivirus I*	Aydin-like pestivirus	-	*Ovis* spp., *Capra* spp., *Bos taurus*	04-TR	NC018713	12,292	[11]
*Pestivirus J*	rat pestivirus	NrPV	*Rattus norvegicus*	NYC-D23	NC025677	12,983 ^2^	[20]
*Pestivirus K*	atypical porcine pestivirus	APPV	*Sus scrofa*	515	NC038964	11,276 ^2^	[14]

cp: cytopathic; ^1^ Virus names and virus abbreviations are not official ICTV designations. Given is the first proposed or commonly used abbreviation; ^2^ complete polyprotein encoding sequence.

**Table 2:** GenBank accession numbers of reference sequences.

Species	GenBank Acc. No. of Virus Sequences Used
*Pestivirus A*	JN400273, AF526381, KX987157, M96751, AB078950, LT631725, KF896608, KP313732, KX577637, KP941591, JQ799141, KC757383, LC089876, KC853441
*Pestivirus B*	LC006970, KT875169, FJ527854, GQ888686, KX096718, KJ000672, AB567658, AF002227, KT832818, HQ258810, JF714967
*Pestivirus C*	X87939, AY646427, KF669877, J04358, KC851953, AF407339, KJ619377, FJ529205, GQ923951, KU504339, KP233070, KM362426
*Pestivirus D*	AF037405, AB897785, U70263, KJ463422, KC963426, KF918753, GU270877, KF925348, AF144618
*Pestivirus E*	AY781152
*Pestivirus F*	EF100713
*Pestivirus G*	AF144617, KJ660072
*Pestivirus H*	FJ040215, KC297709, JX469119, JX985409, AB871953, KC788748, HQ231763, JQ612704
*Pestivirus I*	KM408491, JX428945
*Pestivirus J*	KJ950914
*Pestivirus K*	KU041639, KX77872, KR011347, KX929062, KU194229, LT594521, MN099169, MH885413, MH499646, MH307700, KY475593, MH499642, MH493896, MK216752

**Table 3:** Recently reported pestivirus sequences.

Proposed Species	Virus Name	Abbreviation ^1^	Host Species	Isolates	Available Sequences			Ref.
					GenBank	Region	Length [b]	
*Pestivirus L*	Linda virus	LindaV	*Sus scrofa*	Austria1	NC035432	genome	12,614	[40]
*Pestivirus M*	Phocoena pestivirus	PhoPeV	*Phocoena phocoena*	PhoPeV-1	MK910227	genome	11,880	[22]
				PhoPeV-2	MK910228	genome	11,880	[22]
				PhoPeV-3	MK910229	genome	12,060	[22]
*Pestivirus N*	Tunisian sheep-like pestivirus	TSV	*Capra aegagrus hircus*	70292/2007/EN	MZ664273	cds	12,268	[39]
			*Ovis gmelini aries*	92019/2007/AG	MZ664274	genome	12,286	[39]
*Pestivirus O*	ovine/IT pestivirus	ovIT PeV	*Ovis gmelini aries*	338710-2/2017	MK618725	partial	11,143	[41]
				338710-3/2017	MK618726	cds	12,173	[41]
				1756/2017	MG770617	cds	12,173	[41]
*Pestivirus P*	pangolin pestivirus	DYPV	*Amblyomma javanense*	DYAJ1	MK636874	genome	12,443	[21]
			*Manis javanica*	DYCS	MK636875	genome	12,446	[21]
*Pestivirus Q*	rodent pestivirus	RtNn-PeV	*Niviventer niviventer*	HuB2014	KY370101	cds	13,220	[24]
*Pestivirus R*	rodent pestivirus	RtAp-PeV	*Apodemus peninsulae*	JL2014	KY370100	cds	12,768	[24]
n.a.	rodent pestivirus	RtNe-PeV	*Niviventer excelsior*	SC2014	KY370099	partial	11,644	[24]
n.a.	rodent pestivirus	RtAd-PeV	*Apodemus draco*	SAX2015	KY370102	partial	11,551	[24]
n.a.	rodent pestivirus	RtNn-PeV	*Niviventer niviventer*	SAX2015	KY370103	partial	11,435	[24]
*Pestivirus S*	bat pestivirus	BtSk-PeV	*Scotophilus kuhlii*	1/GX2017	MH282908	cds	11,921	[23]
n.a.	bat pestivirus			3/GX2017	MH282910	partial	7266	[23]
n.a.	bat pestivirus			4/GX2017	MH282911	partial	7132	[23]

n.a., not assigned (no complete cds available); ^1^ Virus names and virus abbreviations are not official ICTV designations. Given is the first proposed or commonly used abbreviation.

**Table 4:** Maximum p-distances between members of individual pestivirus species.

Species	Virus Name	p-Distances		
		CDS	Polyprotein	NS5B_3312–3837_
*Pestivirus A*	BVDV-1	<0.22	<0.15	<0.11
*Pestivirus B*	BVDV-2	<0.17	<0.11	<0.10
*Pestivirus C*	CSFV	<0.19	<0.13	<0.07
*Pestivirus D*	BDV	<0.24	<0.15	<0.13
*Pestivirus H*	HoBi	<0.10	<0.07	<0.05
*Pestivirus K*	APPV	<0.20	<0.10	<0.07

**Table 5:** Minimum p-distances between members of selected pestivirus species.

Species Compared		Minimum p-Distances		
		CDS	Polyprotein	NS5B_3312–3837_
*Pestivirus A*	*Pestivirus B*	>0.30	>0.24	>0.17
*Pestivirus C*	*Pestivirus D*	>0.29	>0.21	>0.14
*Pestivirus C*	*Pestivirus I*	>0.28	>0.20	>0.14
*Pestivirus D*	*Pestivirus I*	>0.28	>0.19	>0.14
*Pestivirus L*	*Pestivirus F*	0.35	0.31	0.22
*Pestivirus M*	*Pestivirus F*, *Pestivirus L*	>0.37	>0.36	>0.22
*Pestivirus N*	*Pestivirus C*	>0.24	>0.16	>0.09
*Pestivirus N*	*Pestivirus I*	>0.26	>0.18	>0.13
*Pestivirus N*	*Pestivirus O*	0.23	>0.14	0.11
*Pestivirus O*	*Pestivirus C*	>0.22	>0.14	>0.11
*Pestivirus P*	*Pestivirus F*, *Pestivirus M*	>0.41	>0.47	>0.33
*Pestivirus Q*	*Pestivirus R*	0.40	0.42	0.33
*Pestivirus Q*	*Pestivirus J*	0.30	0.22	0.17
*Pestivirus R*	*Pestivirus Q*	0.40	0.42	0.33
*Pestivirus R*	*Pestivirus J*	0.40	0.42	0.31
*Pestivirus S*	*Pestivirus K*	>0.40	>0.40	>0.29
